# Risk factors of precancerous cervical lesions: The role of women’s socio-demographic, sexual behavior and body mass index in Amhara region referral hospitals; case-control study

**DOI:** 10.1371/journal.pone.0249218

**Published:** 2021-03-26

**Authors:** Birhan Tsegaw Taye, Muhabaw Shumye Mihret, Haymanot Alem Muche

**Affiliations:** 1 Department of Midwifery, College of Medicine and Health Sciences, Debre Birhan University, Debre Birhan, Ethiopia; 2 Department of Clinical Midwifery, School of Midwifery, College of Medicine and Health Sciences, University of Gondar, Gondar, Ethiopia; University of Mississippi Medical Center, UNITED STATES

## Abstract

**Background:**

Cervical cancer remains one of the major public health challenges in low and middle-income countries including Ethiopia. There was a scarce of evidence regarding the effect of woman’s socio-demographic characteristics and body mass index on the development of precancerous cervical lesions in Ethiopia. Therefore, the current study aimed at identifying the risk factors of precancerous cervical lesions among women visiting referral hospitals for cervical cancer screening in Amhara national regional state.

**Methods:**

A hospital-based case-control study was conducted from 22 December 2019 to 8 April 2020 among 200 women including 67 visual inspections with acetic acid (VIA) positive women (i.e., cases) and 133 visual inspections with acetic acid (VIA) negative women (i.e., controls). The study was conducted at randomly selected referral hospitals in Amhara national regional state. Data were collected mainly through face to face interview and chart review using structured questionnaire and checklist respectively. Data were then entered to EpiData version 4.6 and exported to SPSS version 25 for analysis. Binary logistic regression model was fitted and variables with p-value of < 0.2 at bivariable logistic regression analysis were candidates for the multivariable analysis. Level of significance was claimed based on adjusted odds ratio (AOR) with 95% confidence interval (CI) at p-value of ≤ 0.05.

**Results:**

This study illustrates that the odds of being positive for precancerous cervical lesion (PCL) were higher among women who had body mass index (BMI) of <18.5 kg/m2 (AOR = 3.83; 95% CI: 1.26, 8.76), early coitarche (AOR = 3.15; 95% CI: 1.50, 11.49, history of using oral contraceptive pills (AOR = 2.74; 95% CI: 1.6, 7.4), lifetime sexual transmitted infections (AOR = 3.73; 95% CI: 2.5, 12.28) and multiple sexual partners (AOR = 3.23; 95% CI: 1.82, 9.29). On the other hand, participants’ BMI of ≥25 kg/m2 (AOR = 0.46; 95% CI: 0.36, 0.75) and level of education of college and above (AOR = 0.29; 95% CI: 0.23, 0.77) were identified to be protective factors of PCL.

**Conclusion:**

Most of the determinants of precancerous cervical lesions were modifiable and mainly related to women’s socio-demographic characteristics, sexual behaviors and body mass index. Therefore, strengthening awareness on safe sexual practices and healthy life styles through information, education and communication (IEC), and behavioral change communication (BCC) would decrease the incidence of precancerous cervical lesions.

## Introduction

The precancerous cervical lesion (PCL) is a pre-invasive stage of cervical cancer and is preceded by abnormal changes in the cells of the cervix or neck of the uterus [1,2]. It is mainly caused by a sexually transmitted virus called human papilloma virus (HPV). HPV poses different genotypes of which HPV type 16 and 18 are the known high-risk genotypes in bringing about PCL and detected at about 90% of the women with squamous cervical cell carcinoma [3,4]. Evidence suggests that secondary factors or HPV cofactors could facilitate the progress of HPV infections to PCL [5]. Preventing abnormal cervical cell change prior to its advancement to PCL from the very beginning is one of the cost-effective and primary preventive approaches of cervical cancer burdens [6,7].

Cervical cancer is the third most common malignancy among women worldwide and it remains a leading cause (25.2%) of the cancer-related deaths in developing countries [8]. In low and middle-income countries (LMIC), its incidence is nearly twice and its death rates is about three times higher compared with respective indicators in high-income countries [9]. Sub-Saharan African (SSA) nations, particularly East African countries carry the highest-burden which accounts for about 23.3% of morbidities and 16.54% of mortalities [10,11]. The overall burden of cervical cancer is projected to rise worldwide ranging from 2.4% to 25.2% [8,12].

In Ethiopia, women are tragically dying of cervical cancer due to the silent nature of the disease until it becomes quite advanced and the presence of large gaps on responsible factors [11,13,14]. It is the second most frequent cancer among women next to breast cancer. Even though the cause of PCL has been the subject of several studies, factors associated with PCL remain poorly documented [9,12,15–19]. The 2018 International Agency for Research on Cancer (IARC) and Global Cancer Observation (GLOBOCAN) data show that Ethiopia is still faced with an increase in the absolute number of cases being diagnosed and requiring treatment [8,9]. The frequency of PCL was about 6.7–22.1% and 15.9% across the country and the Amhara national regional state respectively, considerably higher among HIV-positive women [20].

To address this significant public health issue; Ethiopia adopted the United Nations (UN), Sustainable Development Goal (SDG) and World Health Organization (WHO) Global Action Plan and designed the National Cancer Control Plan (NCCP). WHO recommends that early identification of premalignant lesions through visual inspection with acetic acid (VIA) and application of “screen and treat” approach can prevent invasive cervical cancer in high-risk women, and this approach is endorsed and applied by Ethiopian Federal Ministry of Health (FMOH) [4,21–25]. Nowadays, achieving SDG 3.4 in the reduction of premature mortality from Non-Communicable Diseases (NCD) such as cervical cancer by 2030 is becoming a considerable government concern. Despite these promising commitments, cervical cancer continues to be a preventable leading cause of death among women [6,25]. Recognizing the risk factors of PCL could have a supreme imperative merit for designing more targeted primary prevention approaches and screening programs to reduce the burden of cervical cancer. Factors associated with PCL can be categorized as socio-demographic, reproductive lifestyle and sexual behavior related factors [14].

Existing evidence suggests that persistent utilization of oral contraceptive pills (OCPs) is a risk factor for PCL [20]. In Ethiopia, OCPs utilization has steadily increased over the last 15 years from 6% in 2000 to 35% in 2016. By method, the largest growth of contraceptive utilization among women has been observed in injectable (medroxyprogesterone acetate) use which has expanded from 3% in 2000 to 23% in 2016 followed by implant which has grown from less than 1% in 2000 to 8% in 2016 [26].

The effect of body mass index (BMI) on the PCL is not much clear [27]. There are claims that underweight women might have increased risk developing PCL while overweight women have decreased risk of experiencing PCL as compared to women with normal BMI [28]. However, the exact effect of BMI on PCL remains less known [29].

Documentation of key risk factors of PCL is compulsory for interventions targeting on prevention of cervical cancer. In this regard, researches in Ethiopia have increased in number steadily over the past decade. However, these studies primarily focused on the screening uptake, advanced cancer, and the proportion of the condition. Thus, there was scarce of evidence on the role of women’s socio-demographic, body mass index, and sexual habits on PCL development. In this context, having the knowledge of key risk factors of PCL in the first instance may contribute to the prevention of cervical cancer. Therefore, the main aim of this study was to fill these gaps and to provide tangible information on the predictors of PCL among women screened for cervical cancer.

## Material and methods

### Study setting and period

A hospital-based case-control study was conducted from 22 December 2019 to 8 April 2020. The study was conducted at referral hospitals in Amhara national regional state, northern Ethiopia. According to the Ethiopian Central Statistics Agency, the region has a projected population of 21.5 million people with a fifty-fifty numerical split between the sexes. The region has about 82 hospitals (including 6 referral hospital, 3 general hospitals and 73 primary hospitals at the time of study), 847 health centers, and 3,342 health posts. Also, each referral hospital is assumed to serve about 5.2 million people, has 200–400 beds, documents an average of around 1488 precancerous cases annually. The six referral hospitals found in the region are University of Gondar referral hospital, Debre Markos referral hospital, and Debre Birhan referral hospital, Felegehiwot referral hospital, Tibebe Gion referral hospital and Dessie referral hospital.

### Sampling procedure

Three of the 6 referral hospitals were randomly selected. These are University of Gondar referral hospital, Debre Markos referral hospital and Debre Birhan referral hospital and these hospitals regularly provide VIA screening services for all 21-49-year-old women. The total sample size was allocated to each selected referral hospital proportionally based on the average monthly client flow reviewed from a four-month registration book. The total population based on the last four-month report was 266 (i.e. 79 cases and 187 controls).

We included 21-49-year-old women who have been screened for cervical cancer in the study settings during the data collection period. Eligible study participants were recruited among those women who were screened with VIA. In this aspect, PCL positive was identified when an aceto-whitish lesion with well-defined margins observed within the vicinity of the transformation zone if the whole cervix turned white by VIA screening while PCL negative was labeled when there is no aceto-whitish lesion on the surface of the cervix by VIA screening [2]. Hence, PCL positive women were enrolled as cases, whereas PCL negative women were regarded as controls. All PCL positive and negative women were included in the study until the required sample size was obtained. Diagnostic criteria were as per cervical cancer prevention guideline for low-resource settings, and screening results were defined as: VIA positive: presence of raised and thickened white plaques or acetowhite epithelium, usually near the Squamo-columnar junction (SCJ). VIA negative: presence of smooth, pink, uniform and featureless cervix; cervical ectropion; polyp; cervicitis; inflammation; and/or nabothian cyst after applying a dilute solution of acetic acid [13].

### Sample size determination

The sample size for the study was determined using Epi info version 7 software, by considering the following assumptions; the level of significance—95%, power—80%, ratio of cases to controls—1: 2, the proportion of controls exposed—36.68%, Odds Ratio (OR) - 2.55, percent of cases with exposure 61.4% from a previous similar study done in Addis Ababa [2]. Thus, the minimum adequate sample size for this study was obtained to be 183. By considering a 10% non-response rate, the final sample size turned to be 202 individuals (i.e., 67 cases and 135 controls).

### Data collection tool and procedures

Data were collected through face to face interview and chart review by using a structured questionnaire and checklist respectively. In addition, weight and height were measured objectively to determine the women’s BMI. The questionnaire was first prepared in English language, translated into the local (i.e., Amharic) language and then back to English language to keep its consistency. Six health care personnel were recruited for data collection process. These include three BSc midwives, who were working at VIA screening unit, for data collection, and three MSc midwives for supervision.

### Data processing and analysis

Data were checked, coded, entered into EpiData version 4.6 and then exported to SPSS version 25 for analysis. Both descriptive and analytical procedures were undertaken. Descriptive statistics like frequencies and cross-tabulations were then performed and the results were presented in texts and tables. Binary logistic regression model was fitted and variables with p-value of < 0.2 in the bivariable logistic regression analysis were candidates for the final model analysis. Level of significance was declared based on the adjusted odds ratio (AOR) with 95% confidence interval (CI) at p-value of ≤ 0.05.

### Ethical considerations

The ethical clearance was obtained from the Institutional Review Board (IRB) of the University of Gondar. A formal letter of cooperation was written to each referral hospital from the Amhara regional health bureau and the permission of hospitals was obtained from hospital administrators. Written permission letter was also received from hospital managers and ward coordinators in the study settings. Before data collection started, the study participants were informed about the objective and purpose of the study (see S1 File). They were also told that their participation would purely voluntary and keep strictly confidential and used for only research purposes. Then written informed consent was obtained from each study participant individually. During data collection, positive for PCL respondents were received appropriate counseling and care by the data collectors.

## Result

### Socio-demographic characteristics of the study participants

Two hundred two participants were enrolled in this study. However, two women from the control group refused to participate and this brought about a response rate of 99%. Forty-six (68.7%) of the cases and 71 (53.7%) of the controls were found in the age group of 40–49 and 30–39 years respectively. The participants’ mean age (±Standard Deviation (SD)) was 40.1 (±7.26) years for the cases and 36.0 (±6.80) years for the controls. Thirty-five (52.2%) of the cases and 98 (73.7%) of the controls were married, of whom 28 (41.8%) of the cases and 70 (52.6%) of the controls were housewives. About 44 (65.7%) of the cases and 62 (46.6%) of the controls had no formal education, whereas 11 (16.5%) of the cases and 43 (32.3%) of the controls attained higher education (**Table 1**).

**Table 1:** Socio-demographic characteristics of the study participants in Amhara region referral hospitals, 2019/20 (n = 200).

Variable	Case n (%)	Control n (%)
**Age (in years)**		
21–29	9(13.4)	24(18)
30–39	12(17.9)	71(53.4)
40–49	46(68.7)	38(28.6)
**Respondent educational status**		
No formal education	44(65.7)	62(46.6)
Primary education	7(10.4)	14(10.5)
Secondary education	5(7.5)	14(10.5)
College or above	11(16.4)	43(32.4)
**Marital status**		
Married	35(52.2)	98(73.7)
Single	2(3)	6(4.5)
Widowed	4(6)	7(5.3)
Divorced/separated	26(38.8)	22(16.5)
**Respondent occupation**		
Housewife	28(41.8)	70(52.6)
Merchant	8(11.9)	9(6.8)
Self-employed	20(29.9)	7(5.3)
Government/private employee	11(16.4)	47(35.4)
**Partner/husband educational status (n = 151)**		
No formal education	27(57.4)	42(40.4)
Primary education	4(8.5)	10(9.6)
Secondary education	7(14.9)	14(13.5)
College or above	9(19.2)	38(36.5)
**Partner/husband occupation (n = 151)**		
Farmer	12(17.9)	33(24.8)
Merchant	4(6)	13(9.7)
Daily laborer	6(9)	11(8.3)
Government/private employee	13(19.4)	36(27.1)
Self-employed	12(17.9)	11(8.3)
Others†	20(29.8)	29(21.8)
**Average family monthly income (in ETB)**		
≤1000	15(20.8)	13(9.8)
1001–3000	31(46.3)	77(57.9)
3001–4999	19(28.4)	35(26.3)
≥5000	3(4.5)	8(6)
**Residence**		
Rural	26(38.8)	50(37.6)
Urban	41(61.2)	83(62.4)
**Religion**		
Orthodox	64(95.5)	129(97)
Muslim	3(4.5)	4(3)

† single, divorced, and widowed.

### Reproductive health-related characteristics of the study participants

The foremost contraceptive method used by participants was injectable followed by OCPs. The extent of contraceptive use was 103 (77.4%) and 49 (73.1%) among the cases and the controls respectively. OCPs had been utilized by 13% of the cases and 32.8% of the controls. The mean menarche age was 14.93 (SD: ±1.24) for the cases and 15.08 (SD: ±1.13) for the controls. Among the respondents, 85.1% of the cases and 94.7% of the controls had a history of childbirth. The median parity of both cases and controls was 3 (Inter Quartile Range (IQR) = 2–5) **(Table 2**).

**Table 2:** Reproductive health-related characteristics of study participants in Amhara region referral hospitals, 2019/20 (n = 200).

Variable	Case n (%)	Control n (%)
**Ever use contraceptive**		
Yes	49(73.1)	103(77.4)
No	18(26.9)	30(22.6)
**Use oral contraceptive pills**		
Yes	22(32.8)	18(13.5)
No	45(67.2)	115(86.5)
**Duration of oral contraceptive used n = 40**		
<5years	15(68.2)	14(77.8)
≥5years	7(31.8)	4(22.2)
**Use injectable** (medroxyprogesterone acetate)		
Yes	23(34.3)	59(44.4)
No	44(65.7)	74(55.6)
**Duration of injectable used n = 82**		
<5years	18(78.3)	45(76.3)
≥5years	5(21.7)	14(23.7)
**Use implant**		
Yes	6(9)	25(18.8)
No	61(91)	108(81.2)
**OTHER**†		
Yes	10(14.9)	17(12.8)
No	57(85.1)	116(87.2)
**Using contraceptive currently n = 48**		
Yes	14(20.9)	34(25.6)
No	53(79.1)	99(74.4)
**Currently using contraceptive type (n = 48)**		
Pills	6(43)	6(17.6)
Injectable	5(35.7)	14(41.2)
Implant	1(7.1)	9(26.5)
IUCD	1(7.1)	3(8.8)
Others†	1(7.1)	2(5.9)
**Age at menarche in years**		
≤12	2(3)	2(1.5)
13–14	17(25.4)	34(25.6)
≥15	48(71.6)	97(72.9)
**Ever use condom**		
Ever	12(17.9)	16(12)
Never	55(82.1)	117(88)
**Ever pregnant**		
Yes	57(85.1)	126(94.7)
No	10(14.9)	7(5.3)
**Parity (n = 179)**		
1–3	29(50.9)	69(56.6)
≥4	28(49.1)	53(43.4)
**Age at first birth (in years) (n = 179)**		
<20	33(57.9)	73(59.8)
≥20	24(42.1)	49(40.2)
**Mode of delivery (n = 179)**		
Spontaneous/assisted vaginal	50(89.3)	115(93.5)
C/S delivery	6(10.7)	8(6.5)
**Average birth interval (in a month) (n = 153)**		
≤24	20(38.5)	41(40.6)
25–36	20(38.5)	39(38.6)
≥37	12(23)	21(20.8)
**History of abortion**		
Yes	20(29.9)	31(23.3)
No	47(70.1)	102(76.7)
**Type of abortion (n = 51)**		
Spontaneous	9(45)	26(83.9)
Induced	11(55)	5(16.1)
**Abortion management (n = 51)**		
Abort by itself	7(35)	22(71)
Took tablet	3(15)	7(22.5)
MVA	10(50)	2(6.5)
**Family history of cervical cancer**		
Yes	4(6)	6(4.5)
No	63(94)	127(95.5)

† = Natural contraceptive, IUD, condom, and post-pill.

MVA = Manual Vacuum Aspiration.

### BMI and sexual behavior characteristics of the participants

About 31.3% of the cases and 21.8% of the controls had been screened for cervical cancer before the index screening. The median BMI of the cases and the controls was 21 and 23.47 respectively. Whereas, the median coitarche age of the cases and the controls was 15 and 15 years respectively. Likewise, the proportion of early sexual activity (i.e., coitarche <15 years) was 38.8% of the cases and 18% of the controls. Thirty (44.8%) of the cases and 23 (17.3%) of the controls had a history of diagnosed lifetime sexual transmitted infections (STI). All the participants were tested for Human Immune Deficiency Virus/Acquired Immune Deficiency Disease (HIV/AIDS) with 11(16.4%) of the cases and nine (6.8%) of the controls were positive for the disease. Median of number of lifetime sexual partners was two (IQR = 1–3) for the cases and one (IQR = 1–2) for the controls respectively. Forty-eight (71.8%) of the cases had ≥2-lifetime sexual partners, whereas more than half (60.9%) of the controls had only one sexual partner (**Table 3**).

**Table 3:** Lifestyle and sexual behavior characteristics of respondents in Amhara region referral hospitals, 2019/20 (n = 200).

Variable	Case n (%)	Control n (%)
**Ever screen for cervical cancer**		
Yes	21(31.3)	29(21.8)
No	46(68.7)	104(78.2)
**Last screen result for PCL**		
Positive	17(81)	1(3.4)
Negative	4(19)	28(96.6)
**Ever history of Cigarette smoking**		
Yes	2(3)	0
No	65(97)	133(100)
**BMI**		
18.5–24.99	43(64.2)	91(68.4)
<18.5	17(25.4)	7(5.3)
≥25	7(10.4)	35(26.3)
**Age at Coitarche (in years)**		
<15	26(38.8)	24(18)
15–17	20(29.9)	49(36.8)
≥18	21(31.3)	60(45.2)
**Age at first marriage (in years), n = 192**		
<18	48(73.8)	65(51.2)
≥18	17(27.2)	62(38.8)
**Participant lifetime history of STI**		
Yes	30(44.8)	23(17.3)
No	37(55.2)	110(82.7)
**Timing of diagnosis of STI n = 52**		
>5 years	22(73.3)	7(31.8)
≤5 years	8(26.7)	15(68.2)
**Partner/husband lifetime STI**		
No	41(61.2)	89(66.9)
Yes	20(29.8)	34(25.6)
Don’t know	6(9)	10(7.5)
**Participant history of lifetime genital ulcer**		
Yes	22(32.8)	21(15.8)
No	45(67.2)	112(84.2)
**Partner/husband history of lifetime genital ulcer or swelling**		
No	439(64.2)	93(69.9)
Yes	12(17.9)	14(10.6)
Don’t know	12(17.9)	26(19.5)
**HIV test result**		
Positive	11(16.4)	9(6.8)
Negative	56(83.6)	124(93.2)
**Start ART, n = 20**		
Yes	9(81.8)	8(88.9)
No	2(18.2)	1(11.1)
**Participant number of lifetime sexual partners**		
One	19(28.4)	81(60.9)
Two and above	48(71.6)	52(39.1)
**Timing of sexual partner encountered n = 80**		
>5 years	20(55.6)	23(52.3)
≤5 years	16(44.4)	21(47.7)
**Partner/husband other numbers of lifetime sexual partners**		
No	25(37.3)	80(60.2)
One	4(6)	13(9.8)
Two and above	33(49.2)	26(19.5)
Don’t know	5(7.5)	14(10.5)

### Risk factors of precancerous cervical lesion

Both bivariable and multivariable logistic regression analyses have been carried out and variables with P-value of <0.2 in the bivariable analysis and had no missed values were candidates for the final model. The result of multivariable logistic regression analysis showed that participant’s BMI of <18.5 kg/m2 and ≥25 kg/m2, college and above level of education, age at coitarche <15, use of oral contraceptive pills, history of lifetime STI, and lifetime multiple sexual partners had a statistically significant association with PCL.

Accordingly, the risk for PCL was higher among women with BMI of <18.5 kg/m2 (AOR = 3.83,95% CI:1.26, 8.76) and lower among women with BMI ≥ 25 kg/m2 (AOR = 0.46, 95% CI: 0.36, 0.75) than normal-weight women. The likelihood of having PCL was 71% times lower among women who attend college and above level of education than their counterparts (AOR = 0.29; 95%CI: 0.23, 0.77). Similarly, the odds of having PCL among women who had a history of sexual intercourse before the age of 15 were four times higher than those women who initiated coitarche in the older age (i.e. ≥18years) (AOR = 3.15; 95% CI: 1.5, 11.49). Likewise, participants who had history of OCPs utilization were 2.74 times more likely to be positive for PCL than non-users (AOR = 2.74; 95% CI: 1.6, 7.4). Moreover, those women who had any lifetime STI were 3.73 times more likely to get PCL as compared to the reference group (AOR = 3.73; 95% CI: 2.5, 12.28). Also, the odds of having PCL were three-fold higher among women who have multiple sexual partners (AOR = 3.23; 95% CI: 1.82, 9.29) (**Table 4**).

**Table 4:** Bivariable and multivariable analysis of selected variables on determinants of PCL study participants in Amhara region referral hospitals, 2019/20 (n = 200).

Variable	Case n(%)	Control n(%)	COR(95%CI)	AOR(95%CI)
**Age (in years)**				
21–29	9(13.4)	24(18)	1.0	
30–39	12(17.9)	71(53.4)	0.45(0.17, 1.2	0.4(0.1, 1.4)
40–49	46(68.7)	38(28.6)	3.23(1.34, 7.8)	2.97(1.0, 13.4)
**Participant level of education**				
No formal education	44(65.7)	62(46.6)	1.0	
Primary education	7(10.4)	14(10.5)	1.96(0.64, 6.01	0.78(0.09, 3.6)
Secondary education	5(7.5)	14(10.5)	1.4(0.41,4, 72)	0.46(0.06, 2.16)
College or above	11(16.4)	43(32.4)	0.36(0.31,0.68)	0.29(0.23, 0.77)**
**Partner/husband occupation**				
Farmer	12(17.9)	33(24.8)	1.0	
Merchant	4(6)	13(9.7)	0.85(0.23, 3.1)	0.55(0.09, 3.23)
Daily laborer	6(9)	11(8.3)	1.5(0.45, 4.95)	0.60(0.1–3, 55)
Gov’t/private employee	13(19.4)	36(27.1)	1.0(0.4, 2.48)	0.56(0.15, 2.1)
Self-employed	12(17.9)	11(8.3)	3.0(1.05, 8.59)	1.83(0.45, 7.47)
Others†	20(29.8)	29(21.8)	1.9(0.79, 4.54)	0.70(0.2, 2.53)
**BMI**				
18.5–24.99	43(64.2)	91(68.4)	1.0	
<18.5	17(25.4)	7(5.3)	5.14(1.2, 13.2)	**3.83(1.26, 8.76)** *
≥25	7(10.4)	35(26.3)	0.42(0.4, 0.71)	0.46(0.36, 0.75)*
**Age at Coitarche**				
<15 years	26(38.8)	24(18)	3.1(1.47, 6.52)	**3.15(1.5, 11.49)** **
15–17 years	20(29.9)	49(36.8)	1.17(0.57, 2.4)	1.17(0.57, 2.39)
≥18 years	21(31.3)	60(45.2)	1.0	
**Use oral contraceptive pills**				
Yes	22(32.8)	18(13.5)	3.1(1.53, 6.37)	**2.74(1.6, 7.4)** *
No	45(67.2)	115(86.5)	1.0	
**Participant history of lifetime STI**				
Yes	30(44.8)	23(17.3)	3.88(2.01, 7.50)	3.73(2.5, 12.28)**
No	37(55.2)	110(82.7)	1.0	
**Participant history of lifetime genital ulcer/swelling**				
Yes	22(32.8)	21(15.8)	2.61(1.31, 5.2)	1.11(0.37, 3.34)
No	45(67.2)	112(84.2)	1.0	
**HIV test result**				
Positive	11(16.4)	9(6.8)	2.71(1.06, 6.9)	2.02(0.5, 8.21)
Negative	56(83.6)	124(93.2)	1.0	
**Partner/husband other numbers of lifetime sexual partners**				
No	25(37.3)	80(60.2)	1.0	
One	4(6)	13(9.8)	0.98(0.29, 3.29)	0.5(0.1, 2.79)
Two and above	33(49.2)	26(19.5)	4.06(2.05, 8.05)	2.05(0.7, 6.01)
Don’t know	5(7.5)	14(10.5)	1.14(0.38, 3.49)	0.78(0.19, 3.27)
**Participant number of lifetime sexual partners**				
One	19(28.4)	81(60.9)	1.0	
Two and above	48(71.6)	52(39.1)	3.94(2.9, 7.43)	3.23(1.82, 9.29)**

*p<0.05

**p≤0.001

**†** single, divorced and widowed.

## Discussion

Cervical cancer is one of the public health challenges especially in developing countries. Primary prevention of cervical cancer is far feasible approach than secondary or tertiary management of cervical cancer in LMIC. Identifying the risk factors and designing and applying respective strategies of PCL is among such cost effective approaches [6,7]. In this viewpoint, the current study was conceived to identify the risk factors of PCL. Accordingly, the odds of being positive for PCL were higher among women who had BMI of <18.5 kg/m2, early coitarche, history of using oral contraceptive pills (OCPs), lifetime sexual transmitted infections and multiple sexual partners. On the other hand, participants’ BMI of ≥25 kg/m2 and level of education of college and above were identified to be protective factors of PCL.

One of the risk factors of PCL which is identified in the current study is underweight (i.e., BMI <18.5. kg/m2) Thus, the odds of being positive for PCL were four-folds higher among underweight women than those women with normal BMI. The association could be explained through the possible relationship among low BMI, poor nutritional practice and low level of immunity. Accordingly, participants with low BMI are likely to be malnourished which in turn could compromise their immunity. In addition, the independent significant association between low BMI and PLC could be ascribed to the cervical favorability for visualization and sampling procedures among women with low BMI. This finding is agreed with the study conducted in Thailand [30].

On the other hand, our study revealed that overweight women were 54% more likely to be protected from PCL than those women with normal BMI. However, readers should interpret this finding with cautions as it could be related to measurement errors secondary to failure to detect PCL among overweight women. In this regard, it has to be nice to put in mind that the transformation zone of the cervix is more difficult to be observed in women with elevated BMI. In connection to this, previous studies also stated that overweight women had the lowest risk of PCL and an elevated risk of invasive cervical cancer—suggesting the under-diagnosis of PCL [28]. Though much attention has been paid to the negative health consequences of overweight, little is known about the effect of body weight on the validity and reliability of screening and diagnostic tests. Logically, any risk factor accompanying increased cervical cancer risk would also be anticipated to increase the risk of PCL. Conversely, a reduced risk of PCL should lead to a corresponding reduced risk of cancer. However, existing evidence revealed a conflicting and contradicting findings in this point of view [28]. Therefore, much attention needs to be paid for screening and diagnostic techniques and equipment to ensure that adequate cervical biopsies and visualization could potentially resolve the paradox.

We found a thought-provoking association between participants’ level of education (i.e. college and above) and PCL development. Accordingly, the likelihood of having PCL was 71% times lower among women who attend college and above level of education than their counterparts. In this perspective, study participants were assessed for all socio-demographic characteristics. and about 11 (16.5%) of the cases and 43 (32.3%) of the controls had attained higher education. This might suggest that educational and general health status has a directly proportional relationship [31] which in turn could be directly related to a decision-making power of the women regarding the risk factors and cervical cancer screening service uptake. Moreover, the association might be related to the participants’ educational status and employment opportunity. In this viewpoint, women completing higher level of education have a better opportunity to get an employment at various organizations where they could meet multidisciplinary professionals. Consequently, they would share rich experiences and develop wide range of awareness on health care related information.

The present study’s finding showcases the statistically significant association between coitarche and PCL. Thus, women who had initiated sexual intercourse before the age of 15 were 3.15 times more likely to be positive for PCL as compared to those women who had commenced it after the age of 18. The association is sensible since the cervical membranes of younger women are immature enough to be susceptible to oncogenic agents which may put the girls at higher risk of contracting STIs more particularly HPV infections [5]. Presumably, the first coitus may form the basis for PCL due to sexual insult to the young cervix, and coitarche at a younger age may suggest an association with high-grade lesions. This finding is corroborated by the results of previous studies carried out in Debre Markos [20], Adama [17], Thailand [30] and Nigeria [19]. Thus, efforts need to be immense to bring about behavioral change of the community towards the timing of coitarche and its possible short and long-term health consequences.

The result of the analysis in the current study indicates that use of OCPs is a predisposing factor for PCL. Hence, OCPs users were three times more likely to be positive for PCL as compared to non- users. This could be linked to endogenous hormonal disturbance although the mechanism how utilization of OCPs increases the risk of PCL is unknown. OCPs may increase hormones that aggravate some cells to multiply more than the normal one and this would increase the susceptibility of cervical cells for persistent infection with high-risk HPV types. This result is aligned with previous studies done in Adama [17] and Debre Markos [20]. On the contrary, a study done in Pakistan reported that ever use or longer use of OCPs for more than 5 years is protective against cervical intraepithelial carcinoma [32]. The reason for this discrepancy might be due to the possibility that OCPs non-users would utilize a barrier methods such as condoms which could precipitate the interplay of other risk factors of PCL [32].

The current study found that women who encountered lifetime STIs have a higher risk of acquisition of PCL. The possible reason for the association might be due to the fact that HPV is the main cause for both PCL and STI [3,33]. This is consistent with previous findings in Ethiopia [2,17,34] and Uganda [14] which deduced that STI is a risk factor for a premalignant cervical lesion. In contrast, a study undertaken in Nigeria concluded that history of acquiring STI did not influence the acquisition of PCL [19]. The possible explanation might be connected to variation in degrees of awareness and/or health-seeking behaviors towards social costs of STIs. Furthermore, such inconsistent findings could be resulted from discrepancies in prevalence of HPV across the study settings and the difference in the laboratory criteria for PCL screening. In this aspect, study conducted in Nigeria used Pap smear for PCL screening unlike the current study which employed VIA.

Moreover, there is a positive association between multiple sexual partner and PCL. The odds of being positive for PCL were three times higher among women having multiple sexual partners than monogamous women. This association is possibly due to the sexually transmitted nature of HPV, perhaps reflecting a greater likelihood of multiple HPV exposures and thus stochastically to greater opportunity for HPV persistence and progression to PCL [3,35]. This is similar with the findings of previous studies conducted in Ethiopia [2,17,18] and Uganda [35]. The probability of encountering an infected sexual partner increases with respect to the number of sexual partners. Finally, authors would like to recommend that readers shall interpret certain findings of this study with precautions as recall and social desirability biases might be committed.

## Conclusion

This study illustrates that the odds of being positive for PCL were higher among women who had BMI of <18.5 kg/m2, early coitarche, history of using oral contraceptive pills, lifetime sexual transmitted infections and multiple sexual partners. On the other hand, participants’ BMI of ≥25 kg/m2 and level of education of college and were identified to be protective factors of PCL. Most of the predictors of precancerous cervical lesions were modifiable and mainly related to women’s socio-demographic characteristics, sexual behaviors and body mass index. Therefore, strengthening awareness on safe sexual practices and healthy life styles through information, education and communication (IEC), and behavioral change communication (BCC) would be helpful for prevention of PCL.

## Supporting information

S1 File Questionaire. (DOCX)

S2 File Amharic version quaestionaire. (DOCX)

S1 Data (SAV)

## Abbreviations

ART: Anti-Retroviral Therapy
BMI: Body Mass Index
HPV: Human Papilloma Virus
LMIC: Low and Middle-Income Countries
OCPs: Oral Contraceptive Pills
PCL: Precancerous Cervical Lesion
SPSS: Statistical Product for Social Science
STI: Sexual Transmitted Infection
VIA: Visual Inspection with Acetic acid
