# Supplementary material for: Risk factors of precancerous cervical lesions: The role of women’s socio-demographic, sexual behavior and body mass index in Amhara region referral hospitals; case-control study
Source: PLoS One. 2021 Mar 26;16(3):e0249218. doi: 10.1371/journal.pone.0249218 (PMC7997028; doi:10.1371/journal.pone.0249218)
Supplement: S1 File — (DOCX) [file pone.0249218.s001.docx]

Information sheet and consent form

Information sheet

University of Gondar College of Medicine and Health Sciences School of Midwifery information sheet on the ‘’Risk factors of precancerous cervical lesions: The role of women’s socio-demographic, sexual behavior and body mass index in Amhara region referral hospitals; Case-control study.’’

Hello! How are you? My name is __________. I am working in this hospital. Now I am a research team member to be conducted here by a postgraduate student in clinical midwifery at the University of Gondar. The purpose of the study is to identify the determinants of PCL among women screened for cervical cancer in Amhara region referral hospitals. If you agree to participate in the study as a respondent, you will not have any risk in participating in the study except the time you spent during the interview. The study may be advantageous in identifying risk factors for the PCL, so it is important to develop strategies that help to improve the prevention and control methods of cervical cancer. All the genuine information obtained from you will strictly keep confidential, your participation is purely voluntary, and no monetary incentives will be given for your participation in the study. You can withdraw any time during conducting the study, also your participation, non-participation, or refusal to answer questions will not have any effect on your life, and your name will not be recorded on this form. If you have any questions, Mr. Birhan Tsegaw is the contact person. Birhan can be reached through a call at **0912671560**, **Email**-**birhantsegaw@gmail.com**

Are you willing to participate in the interview and stay with us for a few minutes (15-20) now?

[ ] Yes, Go to the next page [ ] No, Thanks! Proceed to next eligible participant

**Note:** Women who undergo screening for the cervical precancerous lesion who are from 21-49-year-old**.**

Consent form

I am informed that my identity and the information I give will be treated confidentially. I have also been informed that I can refuse to participate in the study or not respond to questions if I am not interested. Furthermore, I have been informed that I can stop responding to the questions at any time in the process. I am informed that my participation, non-participation, or refusal to answer questions will not have any effect on my life. I am informed that no financial incentives will be given for my participation in the study. I am also informed that my response will be used to develop strategies that help to improve the prevention and control methods of cervical cancer.

If the study subject agrees to participate in the study, thank her and start the interview.

Participant’s code No.______________ signature ______________ Date__________

Interviewer ‘s name---------------------------Signature-------------------Date---------------------

**Note:** No need to enforcing the clients to be included in the study.

Thank you!

Questionnaire

1. Participant’s code number: ____________

**CASE (women with PCL) CONTROL(women without PCL)**

**Part 1: Socio-demographic characteristics**

| S.No | Question | Response | Skip |
| --- | --- | --- | --- |
| 101 | How old are you? | ___________(completed years) |  |
| 102 | What is your marital status? | 1. Single 2. Married   C. Widowed  D. Divorced  E. Separated |  |
| 103 | What is your level of education? | 1. Can’t read and write 2. able to read and write 3. Primary (1-8) 4. Secondary (9-12) 5. Diploma (technical/vocational 6. Higher (degree and above |  |
| 104 | Educational status of your partner? | A. Can’t read and write   1. Able to read and write 2. Primary (1-8) 3. Secondary (9-12) 4. Diploma or technical/vocational 5. Higher (degree and above |  |
| 105 | What is your current occupation status? | 1. Housewife 2. Merchant 3. Daily laborer 4. Governmental employee 5. Private/NGO employee 6. Self-employed 7. student 8. Others (specify______) |  |
| 106 | Occupation of your partner? | 1. Farmer 2. Merchant 3. Daily laborer 4. Gov’t. employee 5. Private employee 6. Self-employed 7. student 8. Other specify_____ |  |
| 107 | How much is your family average monthly income? | __________ ETB |  |
| 108 | Residence of the women? | 1. Rural 2. Urban |  |
| 109 | What is your religion? | A. Orthodox Christian  B. Muslim  C. Protestant  D. Catholic  E. Others(specify______) |  |

**Part 2: Questions related to reproductive health factors**

| 201 | Have you ever used contraceptives? | 1. Yes B. No | 206 |
| --- | --- | --- | --- |
| 202 | If the answer for Q 201 yes, which type of contraceptive do you use? (you can choose more than one choice) | 1. Pills 2. Injectable 3. Implant 4. IUCD 5. Others(specify __) |  |
| 203 | For how long have you been using contraception? If used, write for each. | ----------------in years |  |
| 204 | Are you currently using oral contraception? | 1. Yes B. No |  |
| 205 | If the answer for Q 204 yes, which type of contraceptive do you use? (you can choose more than one choice) | 1. Pill 2. Injectable 3. Implant 4. IUCD 5. Others (specify ___) |  |
| 206 | Do you use a condom whenever you are having sex? | 1. Always B. Sometimes   C. Never use |  |
| 207 | How old were at your menarche? | _________in years |  |
| 208 | Have you ever-experienced post-coital bleeding? | 1. Yes B. No |  |
| 209 | Have you ever been pregnant? | 1. Yes B. No | 219 |
| 210 | How many times have you been pregnant? | ------------- |  |
| 211 | At what age you gave your first birth? | -------------in years |  |
| 212 | How old were you at your first birth? | ________in year |  |
| 213 | What was the mode of delivery? | 1. SVD 2. Instrumental 3. C/S |  |
| 214 | What is the average birth interval between your births? (if she has two or more births) | -------------in months |  |
| 215 | Have you ever-experienced abortion? | 1. Yes B. No |  |
| 216 | If answer for Q 214 yes, how many times? | ____________ |  |
| 217 | How does abortion start? | 1. Spontaneously 2. Induced |  |
| 218 | what did you do after the abortion started? | Abort by itself  Took tablet  MVA  Herbal medicine  Other |  |
| 219 | Do you have a family (mother or sister) history of cervical cancer? | 1. Yes B. No |  |
| Part 3: Questions related to lifestyle and sexual behavior factors | | | |
| 301 | Have you ever screened for cervical cancer before? | 1. Yes B. No | 304 |
| 302 | If answer for Q 301 yes when did you screened? | ___________ |  |
| 303 | What was the result of that screening test? | A. Positive B. Negative |  |
| 304 | Have you ever smoked in your lifetime? | A. Yes B. No | 308 |
| 305 | If yes, how many cigarettes do you smoke in your lifetime? | 1. <100 2. > 100 |  |
| 306 | Do you smoke currently? | A. Yes B. No |  |
| 307 | How long you have been smoke? | __________in months |  |
| 308 | Weight in Kg? | __________ |  |
| 309 | Height in meter? | __________ |  |
| 310 | How old were you when you first had sex? | __________in years |  |
| 111 | If married, how old were your first marriage? | ______(completed years) |  |
| 312 | Have you diagnosed or treated for STIs in your lifetime by health professionals? | A. Yes B. No | 314 |
| 313 | If yes, when do you diagnosed or treated? | 1. Before 5 years’ back 2. 5 years and after |  |
| 314 | Has your partner diagnosed or treated for STIs in his lifetime by health professionals? | A. Yes B. No  C. Don’t know |  |
| 315 | Do you ever have a history of lifetime genital ulcer/swelling? | A. Yes B. No |  |
| 316 | Does your partner ever have a history of lifetime genital ulcer/swelling? | A. Yes B. No  C. Don’t know |  |
| 317 | HIV test result? (chart review) | 1. Positive B. Negative |  |
| 318 | If positive for HIV in Q 315, did you start antiretroviral therapy? | 1. Yes B. No |  |
| 319 | How many sexual partners have you had in your lifetime? | ___________ |  |
| 320 | Does your partner have other partners? | A. Yes C. Don’t know  B. No |  |
| 321 | If answer for Q 318 yes, how many? | ___________ |  |

Thank you!

consent form (Amharic Version)

መረጃ መስጫ ወረቀት

ጎንደር ዩኒቨርስቲ ህክምናና ጤና ሳይንስ ኮሌጅ የሚድዋይፈሪ ትምህርት ቤት የማህፀን ጫፍ ቕድመ ካንሰር ጠንቆች በተመለከተ በአማራ ክልል ሪፈራል ሆስፒታሎች ለካንሰር በሚመረመሩ ሴቶች ላይ የሚደረግ ጥናት ነው።

ጤና ይስጥልኝ፤ ስሜ -------------------------ይባላል፡፡ እኔ በጎንዳር ዩኒቨርሲቲ የሚድዋይፈሪ ትምህርት ቤት የማስተርስ ድግሪ የሚያጠና ተማሪ ከጎንደር ዩኒቨርሲቲ መምህራን ጋር በመታገዝ በአማራ ክልል ሪፈራል ሆስፒታሎች የማህፀን በር ቅድመ ካንሰር ተያያዥ ምክንያቶች ለማጥናት በተዋቀረው ቡድን ውስጥ አባል ነኝ፡፡ ጥናታችንም ወደ ጤና ተቋማት ለቅድመ ካንሰር ምርምራ በመጡ ሴቶች በመጠየቅ የሚከናወን ነው፡፡ እርሶም በጥናት ቡድን አማካኝነት ጥናቱ ላይ ተሳታፊ እንዲሆኑ ተመርጠዋል፡፡ እርሶ የሚሰጡትን መረጃ ከሌሎች ምንጮች ጋር ተዳምሮ የማህፀን በር ቅድመ ካንሰር ተያያዥ ምክንያቶች ለይቶ ለማወቅ ወይም የሚሻሻልበት ሁኔታ ለመፍጠር ታልሞ የተዘጋጀ ጥናት ነው፡፡ በሂደታችን ውስጥ በጥናቱ ላለመካፈል በማኛውም ወቅት ከወሰኑ በማኛውም ሰዓት መጠይቁን እናቆማለን፡፡ በጥናቱ ውስጥ ላለመካፈል በሚወስኑት ውሳኔ የተነሳ የሚደርስቦት አንዳችም ሁኔታ የለም፡፡ በቃለ መጠይቁ ወቅት የሚሰጡት መረጃዎች ለጥናቱ ዓላማ ብቻ የሚውሉና ሚስጢራዊነቱ ሙሉ በሙሉ የተጠበቀ ነው፡፡ በዚህ መጠይቅ ውስጥ ስሞትንና እርሶን ለመለየት የሚያገለግል ነገር አይጻፍም፡፡ ቃለ መጠየቁ የሚወስድብዎት ግዜ ከ 15-20 ደቂቃ ብቻ ነው፡፡ ግልጽ ያልሆነ ነገር ካለ ሊጠይቁን ይችላሉ፡፡ ማንኛውም ጥያቄ ካሎት የጥናቱ መሪ የሆኑትን አቶ ብርሃን ጸጋው በስልክ ቁጥር **251912671560** : **ኢሜል፡** [**tsegawbirhan2@gmail.com**](mailto:tsegawbirhan2@gmail.com) ማግኘት ይችላሉ፡፡

በቃለ መጠይቁ ተስማምቻለሁ_________ ወደ የስምምነት ቅጽ ይለፉ

በቃለ መጠይቁ አልተስማማሁም _______ አመስግነው በዚህ ያብቁ

**አስታውስ**፤ ተሳታፊዋ የማህፀን በር ቅድመ ካንሰር ምርምራ ያደረገች፣ ከ 21-49 ዕድሜ ክልል ውስጥ፣ መሆን ኣለባቸው ፡፡

ክፍል ሁለት: የስምምነት ቅጽ

ተመራማሪው/ዋ የጥናቱን አላማ በሚገባ ግልጽ በሆነ ቋንቋ አስረድተውኛል፡፡ በዚህም መሰረት የጥናቱን አላማ ስለተረዳሁ ለመሳተፍ መስማማቴን በፊርማዬ አረጋግጣለሁ፡፡

የተሳታፊዋ መለያ ቁጥር ___________________ ፊርማ __________________ ቀን _____________

የመረጃ ሰብሳቢ ስም --------------------------- ፊርማ --------------- ቀን ------------

**አስታውስ፤** ተሳታፊዋ በግድ በጥናቱ እንዲሳተፍ አያስገድዱ፡፡

ስለተባበሩን እናመሰግናለን!

Questionnaire (Amharic version)

የተሳታፊዋ መለያ ቁጥር _______

**የማህፀን በር ቅድመ ካንሰር ያላት [ ] የማህፀን በር ቅድመ ካንሰር የሌላት [ ]**

**ክፍል I. የማህበራዊ: ኢኮኖሚያዊና ዲሞግራፊያዊ ሁኔታዎች**

| ቁጥር | ጥያቄ | ምላሽ | ዝለል |
| --- | --- | --- | --- |
| 101 | ዕድሜ | ________በአመት |  |
| 102 | የጋብቻ ሁኔታዎ? | ሀ. ያላገባች ለ. ያገባች  ሐ. ባሏ የሞተባት መ. የተፋታች  ረ. ተለያይተው የሚኖሩ |  |
| 103 | የትምህርት ደረጃዎ? | ሀ. መፃፍና ማንበብ የማትችል  ለ. ማንበብና መጻፍ የምትችል  ሐ. የመጀመሪያ ደረጃ (1_8) ያጠናቀቀች  መ. ሁለተኛ ደረጃ (9_12) ያጠናቀቀች  Hሰ. ዲፕሎማ# ቴክኒክ እና ሙያ ያጠናቀቀች  ረ. ከፍተኛ (ዲግሪ እና ከዛ በላይ) |  |
| 104 | የትዳር (የፍቅር) ጓደኛዎ የት/ት ደረጃ? | ሀ. መፃፍና ማንበብ የማይችል  ለ. ማንበብና መጻፍ የሚችል  ሐ. የመጀመሪያ ደረጃ (1_8) ያጠናቀቀ  መ. ሁለተኛ ደረጃ (9_12) ያጠናቀቀ  ሰ. ዲፕሎማ# ቴክኒክ እና ሙያ ያጠናቀቀ  ረ. ከፍተኛ (ዲግሪ እና ከዛ በላይ) |  |
| 105 | ስራዎ ምንድን ነው? | ሀ. የቤት እመቤት ሰ. የግል ሰራተኛ  ለ. ነጋዴ ረ. የግል ተዳዳሪ  ሐ. ቀን ሰራተኛ ሠ. ተማሪ  መ. የመንግስት ሰራተኛ ሸ. ሌላ…(ይገለጽ) |  |
| 106 | የትዳር (የፍቅር) ጓደኛዎ ስራ ምንድነው? | ሀ. ገበሬ ሰ. የግል ሰራተኛ  ለ. ነጋዴ ረ. የግል ተዳዳሪ  ሐ. ቀን ሰራተኛ ሠ. ተማሪ  መ. የመንግስት ሰራተኛ ሸ. ሌላ…(ይገለጽ) |  |
| 107 | ወርሃዊ የቤተሰብ ገቢ በአማካይ? | ______በብር |  |
| 108 | የመኖሪያ ቦታዎ? | ሀ. ገጠር ለ. ከተማ |  |
| 119 | ሐይማኖት | ሀ. ኦርቶዶክስ ለ. ሙስሊም  ሐ. ፕሮቴስታንት መ. ካቶሊክ ረ. ሌላ___ |  |

**ክፍል II: ስለ ተዋልዶ ጤና ተዛማጅ ጥያቄዎች**

| 201 | የእርግዝና መከላከያ ተጠቀመው ያውቃሉ? | ሀ. አዎ ለ. አልጠቀምም | 206 |
| --- | --- | --- | --- |
| 202 | የእርግዝና መከላከያ እየተጠቀሙ ከሆነ ወይም ከነበረ የትኛውን ዓይነት ነው የሚጠቀሙት? (ከአንድ በላይ መምረጥ ይቻላል) | ሀ. የሚዋጥ ፒል  ለ. በመርፌ የሚሰጥ  ሐ. በክንድ የሚቀበረውን  መ. በማህጸን ውስጥ የሚቀመጥ  ሰ. ሌላ___ |  |
| 203 | ለምን ያህል ግዜ ተጠቀሙ? (ከአንድ በላይ እየተጠቀሙ ከነበሩ ለሁሉም ይንገሩኝ) | ____________ |  |
| 204 | በአሁኑ ሰአት የእርግዝና መከላከያ ይጠቀማሉ? | ሀ. አዎ ለ. አልጠቀምም |  |
| 205 | በአሁኑ ሰአት የእርግዝና መከላከያ እየተጠቀሙ ከሆነ የትኛውን ዓይነት ነው የሚጠቀሙት? (ከአንድ በላይ መምረጥ ይቻላል) | ሀ. የሚዋጥ ፒል  ለ. በመርፌ የሚሰጥ  ሐ. በክንድ የሚቀበረውን  መ. በማህጸን ውስጥ የሚቀመጥ  ሰ. ሌላ___ |  |
| 306 | ግብረ ስጋ ግንኙነት በሚያደርጉበት ጊዜ ኮንዶም ይጠቀማሉ? | ሀ. ሁልጊዜ ለ. አንድ አንድ ጊዜ  ሐ. ተጠቅሜ አላዉቅም |  |
| 207 | በስንት ዓመትዎ ነው የመጀመሪያውን የወር አበባ ያዩት? | ___________በአመት |  |
| 208 | ከግብረ ስጋ ግንኙነት በኋላ ደም የማየት ነገር አሎት? | ሀ. አዎ ለ. የለኝም |  |
| 209 | አርግዘው ያውቃሉ? | ሀ. አዎ ለ. አላውቅም | 219 |
| 210 | ምን ያህል ጊዜ አርግዘው ያውቃሉ? | _______ |  |
| 211 | ስንት ልጆች ወለዱ (ከ7 ወር በኋላ) | _______ |  |
| 212 | በስንት አመትዎ ነው የመጀመሪያውን ልጅ የወለዱት? | ________በአመት |  |
| 213 | የወሊድ ሁኔታ? | ሀ. በማህጸን  ለ. በመሳሪያ በመታገዝ በማህጸን፤  ሐ. በኦፕራሲዎን |  |
| 214 | በኣማካይ በልጆችዎ መካከል ያለ የእድሜ ልዩነት ስንት ነው? (ሁለት እና ከዛ በላይ ልጅ ከወለደች) | ________በወር |  |
| 215 | ውርጃ ኖሮት ያውቃል? | ሀ. አዎ ለ. አያውቅም |  |
| 216 | አዎ ካሉ ስንት ግዜ? | ___________ |  |
| 217 | ውርጃ እንዴት ጀመሮት? | ሀ. በራሱ ጊዜ ለ. እንድጀምር ተደርጎ ነው (በመድሃኒት፣ ይገለጽ) |  |
| 218 | ውርጃው ከጀመረዎት በኋላ ምን አደረጉ? | ሀ. በራሱ ጊዜ ወረደ  ለ. ኪኒን ወሰድኩ  ሐ. በመሳሪያ ታግዠ ወረደልኝ  መ. ከዕፅዋት የተዘጋጀ መድኃኒት ወሰድኩ  ሐ. ሌላ (ይገለጽ...) |  |
| 219 | በቤተሰብ የማህፀን ካንሰር ያለበት ሰው አለ? | ሀ. አዎ ለ. የለም |  |
| **ክፍል III: ስለ ግል አኗኗር እና ወሲባዊ ባህርያት ጥያቄዎች** | | |  |
| 301 | ከዚህ በፊት የማህፀን ጫፍ ካንሰር ተመርምረው ያውቃሉ? | ሀ. አዎ ለ. አላውቅም | 304 |
| 302 | ለመጨረሻ ግዜ የተመረመሩት መቼ ነው? | ____________ |  |
| 303 | የምርመራው ውጤቱ ምን ነበር? | ሀ. ፖዘቲቭ ለ. ነጋቲቭ |  |
| 304 | ሲጋራ ኣጭሰው ያውቃሉ? | ሀ. አዎ ለ. አላጨስም | 308 |
| 305 | በህይወት ዘመኖ ምን ያክል ሲጋራ አጨሱ? | ሀ. <100 ለ. >100 |  |
| 306 | በኣሁኑ ሰአት ያጨሳሉ? | ሀ. አዎ ለ. አላጨስም |  |
| 307 | አዎ ካሉ ለምን ያህል ግዜ አጨሱ? | __________በወር |  |
| 308 | የሰውነት ክብደት | __________በኪሎግራም |  |
| 309 | የሰውነት ቁመት | __________በሜትር |  |
| 310 | ለመጀመሪያ ጊዜ ግብረ ስጋ ግንኙነት ሲያደርጉ እድሜዎ ስንት ነበር? | ___________በአመት |  |
| 311 | የመጀመሪያ ጋብቻ ሲፈጽሙ እድሜዎት ስንት ነበር? (ያገቡ ከሆነ) | ___________በአመት |  |
| 312 | በባለሙያ የተረጋገጠ የአባላዘር በሽታ አለብዎት ተብለው ወይም ታክመው ያውቃሉ? | ሀ. አዎ ለ. አላውቅም |  |
| 313 | አወ ካሉ፡ መቸ ነው የታከሙት ወይም ምርመራ አድርገው ያወቁት? | ሀ. ከ5 አመት በፊት  ለ, ከ5 አመት ወድህ |  |
| 314 | በባለሙያ የተረጋገጠ ባለቤትዎ ወይም የፍቅር ጓደኛዎ የአባለዘር በሽታ አለበት ተብሎ ያውቃል? | ሀ. አዎ ለ. አያውቅም  ሐ. አላውቅም |  |
| 315 | በራስዎ ብልት አካባቢ ላይ የሚያሳክክ ጠባሳ ወይም ዕብጠት ወጥቶቦት ያውቃል? | ሀ. አዎ ለ. አያውቅም |  |
| 316 | ባለቤትዎ (የፍቅር ጓደኛዎ) ብልት አካባቢ ላይ የሚያሳክክ ጠባሳ ወይም ዕብጠት ወጥቶበት ያውቃል? | ሀ. አዎ ለ. አያውቅም  ሐ. አላውቅም |  |
| 317 | የኤች አይ ቪ ምርመራ ውጤት? (ከካርድ የሚዎሰድ) | ሀ. ፖዘቲቭ ለ. ነጋቲቭ | 319 |
| 318 | የምርመራ ውጤት ፖዘቲቭ ከሆነ የኤች አይ ቪ መድሃኒት ጀመረዋል? | ሀ. አዎ ለ. አልጀመርኩም |  |
| 319 | እስከ አሁን ድረስ ከስንት ወንዶች ጋር ግብረ ስጋ ግንኙነት አድርገው ያውቃሉ? | ___________ |  |
| 320 | ባለቤትዎ (የፍቅር ጓደኛዎ) ከሌላ ሰው ጋር ግብረ ስጋ ግንኙነት ኣለው? | ሀ. አለው ለ. የለውም  ሐ. አላውቅም |  |
| 321 | አለው ካሉ ከስንት ሰው? | ___________ |  |

ስለ ሰጡኝ ምላሽ በጣም አመሰግናለሁ!
