# Supplementary material for: Risk factors of precancerous cervical lesions: The role of women’s socio-demographic, sexual behavior and body mass index in Amhara region referral hospitals; case-control study
Source: PLoS One. 2021 Mar 26;16(3):e0249218. doi: 10.1371/journal.pone.0249218 (PMC7997028; doi:10.1371/journal.pone.0249218)
Supplement: S2 File — (DOCX) [file pone.0249218.s002.docx]

Birhan Tsegaw Taye

[tsegawbirhan2@gmail.com](mailto:tsegawbirhan2@gmail.com) (BT)

Corresponding author

consent form (Amharic Version)

መረጃ መስጫ ወረቀት

ጎንደር ዩኒቨርስቲ ህክምናና ጤና ሳይንስ ኮሌጅ የሚድዋይፈሪ ትምህርት ቤት የማህፀን ጫፍ ቕድመ ካንሰር ጠንቆች በተመለከተ በአማራ ክልል ሪፈራል ሆስፒታሎች ለካንሰር በሚመረመሩ ሴቶች ላይ የሚደረግ ጥናት ነው።

ጤና ይስጥልኝ፤ ስሜ -------------------------ይባላል፡፡ እኔ በጎንዳር ዩኒቨርሲቲ የሚድዋይፈሪ ትምህርት ቤት የማስተርስ ድግሪ የሚያጠና ተማሪ ከጎንደር ዩኒቨርሲቲ መምህራን ጋር በመታገዝ በአማራ ክልል ሪፈራል ሆስፒታሎች የማህፀን በር ቅድመ ካንሰር ተያያዥ ምክንያቶች ለማጥናት በተዋቀረው ቡድን ውስጥ አባል ነኝ፡፡ ጥናታችንም ወደ ጤና ተቋማት ለቅድመ ካንሰር ምርምራ በመጡ ሴቶች በመጠየቅ የሚከናወን ነው፡፡ እርሶም በጥናት ቡድን አማካኝነት ጥናቱ ላይ ተሳታፊ እንዲሆኑ ተመርጠዋል፡፡ እርሶ የሚሰጡትን መረጃ ከሌሎች ምንጮች ጋር ተዳምሮ የማህፀን በር ቅድመ ካንሰር ተያያዥ ምክንያቶች ለይቶ ለማወቅ ወይም የሚሻሻልበት ሁኔታ ለመፍጠር ታልሞ የተዘጋጀ ጥናት ነው፡፡ በሂደታችን ውስጥ በጥናቱ ላለመካፈል በማኛውም ወቅት ከወሰኑ በማኛውም ሰዓት መጠይቁን እናቆማለን፡፡ በጥናቱ ውስጥ ላለመካፈል በሚወስኑት ውሳኔ የተነሳ የሚደርስቦት አንዳችም ሁኔታ የለም፡፡ በቃለ መጠይቁ ወቅት የሚሰጡት መረጃዎች ለጥናቱ ዓላማ ብቻ የሚውሉና ሚስጢራዊነቱ ሙሉ በሙሉ የተጠበቀ ነው፡፡ በዚህ መጠይቅ ውስጥ ስሞትንና እርሶን ለመለየት የሚያገለግል ነገር አይጻፍም፡፡ ቃለ መጠየቁ የሚወስድብዎት ግዜ ከ 15-20 ደቂቃ ብቻ ነው፡፡ ግልጽ ያልሆነ ነገር ካለ ሊጠይቁን ይችላሉ፡፡ ማንኛውም ጥያቄ ካሎት የጥናቱ መሪ የሆኑትን አቶ ብርሃን ጸጋው በስልክ ቁጥር **251912671560** : **ኢሜል፡** [**tsegawbirhan2@gmail.com**](mailto:tsegawbirhan2@gmail.com) ማግኘት ይችላሉ፡፡

በቃለ መጠይቁ ተስማምቻለሁ_________ ወደ የስምምነት ቅጽ ይለፉ

በቃለ መጠይቁ አልተስማማሁም _______ አመስግነው በዚህ ያብቁ

**አስታውስ**፤ ተሳታፊዋ የማህፀን በር ቅድመ ካንሰር ምርምራ ያደረገች፣ ከ 21-49 ዕድሜ ክልል ውስጥ፣ መሆን ኣለባቸው ፡፡

ክፍል ሁለት: የስምምነት ቅጽ

ተመራማሪው/ዋ የጥናቱን አላማ በሚገባ ግልጽ በሆነ ቋንቋ አስረድተውኛል፡፡ በዚህም መሰረት የጥናቱን አላማ ስለተረዳሁ ለመሳተፍ መስማማቴን በፊርማዬ አረጋግጣለሁ፡፡

የተሳታፊዋ መለያ ቁጥር ___________________ ፊርማ __________________ ቀን _____________

የመረጃ ሰብሳቢ ስም --------------------------- ፊርማ --------------- ቀን ------------

**አስታውስ፤** ተሳታፊዋ በግድ በጥናቱ እንዲሳተፍ አያስገድዱ፡፡

ስለተባበሩን እናመሰግናለን!

Questionnaire (Amharic version)

የተሳታፊዋ መለያ ቁጥር _______

**የማህፀን በር ቅድመ ካንሰር ያላት [ ] የማህፀን በር ቅድመ ካንሰር የሌላት [ ]**

**ክፍል I. የማህበራዊ: ኢኮኖሚያዊና ዲሞግራፊያዊ ሁኔታዎች**

| ቁጥር | ጥያቄ | ምላሽ | ዝለል |
| --- | --- | --- | --- |
| 101 | ዕድሜ | ________በአመት |  |
| 102 | የጋብቻ ሁኔታዎ? | ሀ. ያላገባች ለ. ያገባች  ሐ. ባሏ የሞተባት መ. የተፋታች  ረ. ተለያይተው የሚኖሩ |  |
| 103 | የትምህርት ደረጃዎ? | ሀ. መፃፍና ማንበብ የማትችል  ለ. ማንበብና መጻፍ የምትችል  ሐ. የመጀመሪያ ደረጃ (1_8) ያጠናቀቀች  መ. ሁለተኛ ደረጃ (9_12) ያጠናቀቀች  Hሰ. ዲፕሎማ# ቴክኒክ እና ሙያ ያጠናቀቀች  ረ. ከፍተኛ (ዲግሪ እና ከዛ በላይ) |  |
| 104 | የትዳር (የፍቅር) ጓደኛዎ የት/ት ደረጃ? | ሀ. መፃፍና ማንበብ የማይችል  ለ. ማንበብና መጻፍ የሚችል  ሐ. የመጀመሪያ ደረጃ (1_8) ያጠናቀቀ  መ. ሁለተኛ ደረጃ (9_12) ያጠናቀቀ  ሰ. ዲፕሎማ# ቴክኒክ እና ሙያ ያጠናቀቀ  ረ. ከፍተኛ (ዲግሪ እና ከዛ በላይ) |  |
| 105 | ስራዎ ምንድን ነው? | ሀ. የቤት እመቤት ሰ. የግል ሰራተኛ  ለ. ነጋዴ ረ. የግል ተዳዳሪ  ሐ. ቀን ሰራተኛ ሠ. ተማሪ  መ. የመንግስት ሰራተኛ ሸ. ሌላ…(ይገለጽ) |  |
| 106 | የትዳር (የፍቅር) ጓደኛዎ ስራ ምንድነው? | ሀ. ገበሬ ሰ. የግል ሰራተኛ  ለ. ነጋዴ ረ. የግል ተዳዳሪ  ሐ. ቀን ሰራተኛ ሠ. ተማሪ  መ. የመንግስት ሰራተኛ ሸ. ሌላ…(ይገለጽ) |  |
| 107 | ወርሃዊ የቤተሰብ ገቢ በአማካይ? | ______በብር |  |
| 108 | የመኖሪያ ቦታዎ? | ሀ. ገጠር ለ. ከተማ |  |
| 119 | ሐይማኖት | ሀ. ኦርቶዶክስ ለ. ሙስሊም  ሐ. ፕሮቴስታንት መ. ካቶሊክ ረ. ሌላ___ |  |

**ክፍል II: ስለ ተዋልዶ ጤና ተዛማጅ ጥያቄዎች**

| 201 | የእርግዝና መከላከያ ተጠቀመው ያውቃሉ? | ሀ. አዎ ለ. አልጠቀምም | 206 |
| --- | --- | --- | --- |
| 202 | የእርግዝና መከላከያ እየተጠቀሙ ከሆነ ወይም ከነበረ የትኛውን ዓይነት ነው የሚጠቀሙት? (ከአንድ በላይ መምረጥ ይቻላል) | ሀ. የሚዋጥ ፒል  ለ. በመርፌ የሚሰጥ  ሐ. በክንድ የሚቀበረውን  መ. በማህጸን ውስጥ የሚቀመጥ  ሰ. ሌላ___ |  |
| 203 | ለምን ያህል ግዜ ተጠቀሙ? (ከአንድ በላይ እየተጠቀሙ ከነበሩ ለሁሉም ይንገሩኝ) | ____________ |  |
| 204 | በአሁኑ ሰአት የእርግዝና መከላከያ ይጠቀማሉ? | ሀ. አዎ ለ. አልጠቀምም |  |
| 205 | በአሁኑ ሰአት የእርግዝና መከላከያ እየተጠቀሙ ከሆነ የትኛውን ዓይነት ነው የሚጠቀሙት? (ከአንድ በላይ መምረጥ ይቻላል) | ሀ. የሚዋጥ ፒል  ለ. በመርፌ የሚሰጥ  ሐ. በክንድ የሚቀበረውን  መ. በማህጸን ውስጥ የሚቀመጥ  ሰ. ሌላ___ |  |
| 306 | ግብረ ስጋ ግንኙነት በሚያደርጉበት ጊዜ ኮንዶም ይጠቀማሉ? | ሀ. ሁልጊዜ ለ. አንድ አንድ ጊዜ  ሐ. ተጠቅሜ አላዉቅም |  |
| 207 | በስንት ዓመትዎ ነው የመጀመሪያውን የወር አበባ ያዩት? | ___________በአመት |  |
| 208 | ከግብረ ስጋ ግንኙነት በኋላ ደም የማየት ነገር አሎት? | ሀ. አዎ ለ. የለኝም |  |
| 209 | አርግዘው ያውቃሉ? | ሀ. አዎ ለ. አላውቅም | 219 |
| 210 | ምን ያህል ጊዜ አርግዘው ያውቃሉ? | _______ |  |
| 211 | ስንት ልጆች ወለዱ (ከ7 ወር በኋላ) | _______ |  |
| 212 | በስንት አመትዎ ነው የመጀመሪያውን ልጅ የወለዱት? | ________በአመት |  |
| 213 | የወሊድ ሁኔታ? | ሀ. በማህጸን  ለ. በመሳሪያ በመታገዝ በማህጸን፤  ሐ. በኦፕራሲዎን |  |
| 214 | በኣማካይ በልጆችዎ መካከል ያለ የእድሜ ልዩነት ስንት ነው? (ሁለት እና ከዛ በላይ ልጅ ከወለደች) | ________በወር |  |
| 215 | ውርጃ ኖሮት ያውቃል? | ሀ. አዎ ለ. አያውቅም |  |
| 216 | አዎ ካሉ ስንት ግዜ? | ___________ |  |
| 217 | ውርጃ እንዴት ጀመሮት? | ሀ. በራሱ ጊዜ ለ. እንድጀምር ተደርጎ ነው (በመድሃኒት፣ ይገለጽ) |  |
| 218 | ውርጃው ከጀመረዎት በኋላ ምን አደረጉ? | ሀ. በራሱ ጊዜ ወረደ  ለ. ኪኒን ወሰድኩ  ሐ. በመሳሪያ ታግዠ ወረደልኝ  መ. ከዕፅዋት የተዘጋጀ መድኃኒት ወሰድኩ  ሐ. ሌላ (ይገለጽ...) |  |
| 219 | በቤተሰብ የማህፀን ካንሰር ያለበት ሰው አለ? | ሀ. አዎ ለ. የለም |  |
| **ክፍል III: ስለ ግል አኗኗር እና ወሲባዊ ባህርያት ጥያቄዎች** | | |  |
| 301 | ከዚህ በፊት የማህፀን ጫፍ ካንሰር ተመርምረው ያውቃሉ? | ሀ. አዎ ለ. አላውቅም | 304 |
| 302 | ለመጨረሻ ግዜ የተመረመሩት መቼ ነው? | ____________ |  |
| 303 | የምርመራው ውጤቱ ምን ነበር? | ሀ. ፖዘቲቭ ለ. ነጋቲቭ |  |
| 304 | ሲጋራ ኣጭሰው ያውቃሉ? | ሀ. አዎ ለ. አላጨስም | 308 |
| 305 | በህይወት ዘመኖ ምን ያክል ሲጋራ አጨሱ? | ሀ. <100 ለ. >100 |  |
| 306 | በኣሁኑ ሰአት ያጨሳሉ? | ሀ. አዎ ለ. አላጨስም |  |
| 307 | አዎ ካሉ ለምን ያህል ግዜ አጨሱ? | __________በወር |  |
| 308 | የሰውነት ክብደት | __________በኪሎግራም |  |
| 309 | የሰውነት ቁመት | __________በሜትር |  |
| 310 | ለመጀመሪያ ጊዜ ግብረ ስጋ ግንኙነት ሲያደርጉ እድሜዎ ስንት ነበር? | ___________በአመት |  |
| 311 | የመጀመሪያ ጋብቻ ሲፈጽሙ እድሜዎት ስንት ነበር? (ያገቡ ከሆነ) | ___________በአመት |  |
| 312 | በባለሙያ የተረጋገጠ የአባላዘር በሽታ አለብዎት ተብለው ወይም ታክመው ያውቃሉ? | ሀ. አዎ ለ. አላውቅም |  |
| 313 | አወ ካሉ፡ መቸ ነው የታከሙት ወይም ምርመራ አድርገው ያወቁት? | ሀ. ከ5 አመት በፊት  ለ, ከ5 አመት ወድህ |  |
| 314 | በባለሙያ የተረጋገጠ ባለቤትዎ ወይም የፍቅር ጓደኛዎ የአባለዘር በሽታ አለበት ተብሎ ያውቃል? | ሀ. አዎ ለ. አያውቅም  ሐ. አላውቅም |  |
| 315 | በራስዎ ብልት አካባቢ ላይ የሚያሳክክ ጠባሳ ወይም ዕብጠት ወጥቶቦት ያውቃል? | ሀ. አዎ ለ. አያውቅም |  |
| 316 | ባለቤትዎ (የፍቅር ጓደኛዎ) ብልት አካባቢ ላይ የሚያሳክክ ጠባሳ ወይም ዕብጠት ወጥቶበት ያውቃል? | ሀ. አዎ ለ. አያውቅም  ሐ. አላውቅም |  |
| 317 | የኤች አይ ቪ ምርመራ ውጤት? (ከካርድ የሚዎሰድ) | ሀ. ፖዘቲቭ ለ. ነጋቲቭ | 319 |
| 318 | የምርመራ ውጤት ፖዘቲቭ ከሆነ የኤች አይ ቪ መድሃኒት ጀመረዋል? | ሀ. አዎ ለ. አልጀመርኩም |  |
| 319 | እስከ አሁን ድረስ ከስንት ወንዶች ጋር ግብረ ስጋ ግንኙነት አድርገው ያውቃሉ? | ___________ |  |
| 320 | ባለቤትዎ (የፍቅር ጓደኛዎ) ከሌላ ሰው ጋር ግብረ ስጋ ግንኙነት ኣለው? | ሀ. አለው ለ. የለውም  ሐ. አላውቅም |  |
| 321 | አለው ካሉ ከስንት ሰው? | ___________ |  |

ስለ ሰጡኝ ምላሽ በጣም አመሰግናለሁ!
